# A long journey into reproducible computational neuroscience

**DOI:** 10.3389/fncom.2015.00030

**Published:** 2015-03-05

**Authors:** Meropi Topalidou, Arthur Leblois, Thomas Boraud, Nicolas P. Rougier

**Affiliations:** ^1^INRIA Bordeaux Sud-OuestBordeaux, France; ^2^Institut des Maladies Neurodégénératives, Université de Bordeaux, UMR 5293Bordeaux, France; ^3^Institut des Maladies Neurodégénératives, Centre National de la Recherche Scientifique, UMR 5293Bordeaux, France; ^4^LaBRI, Université de Bordeaux, Institut Polytechnique de Bordeaux, Centre National de la Recherche Scientifique, UMR 5800Talence, France; ^5^Centre de Neurophysique, Physiologie et Pathologies, Université Paris Descartes, Centre National de la Recherche Scientifique, UMR 8119Paris, France

**Keywords:** reproducible science, publication process, computational models, python language, version control, public repository, notebook

Computational neuroscience is a powerful ally in our quest to understand the brain. Even the most simple model can shed light on the role of this or that structure and propose new hypothesis concerning the overall brain organization. However, any model in Science is doomed to be proved wrong or incomplete and replaced by a more accurate one. In the meantime, for such replacement to happen, we have first to make sure that models are actually reproducible such that they can be tested, evaluated, criticized and ultimately modified, replaced or even rejected. This is where the shoe pinches. If we cannot reproduce a model in the first place, we're doomed to re-invent the wheel again and again, preventing us from building an incremental computational knowledge of the brain.

We have been recently confronted to the problem when we tried to reproduce a model of the literature (Guthrie et al., 2013) concerning a computational model of the basal ganglia. This model was based on a previous modeling study by Leblois et al. (2006) where authors proposed an action selection mechanism based on a competition between the positive feedback, direct pathway through the striatum and the negative feedback, hyperdirect pathway through the subthalamic nucleus. Guthrie et al. (2013) further investigated how multiple level action selection could be performed, and the model has been extended in a manner consistent with known anatomy and electrophysiology of the basal ganglia in the monkey. The model is quite complex, but such is the basal ganglia. We asked authors for the sources of the model only to realize it has been implemented using 6000 lines of Delphi (Pascal language). We were unable to compile it (due to missing packages that we couldn't locate in any repository) and we thus decided to recode it from scratch. Unfortunately, the information provided in the article was not sufficient to allow for the direct reproduction of the model, mainly because there were factual errors in the manuscript and some information was ambiguous or missing. Ultimately, we were able to reach two of the original authors, T. Boraud, and A. Leblois (who are also authors of this commentary) in order to ask them about the details of the model. We joined efforts and proceeded with a complete rewrite, using the Python language (Perkel, 2015), a dedicated library (DANA), a versioning system (git), a public repository (github) and the IPython notebook, merely following the principles of reproducible computational science as proposed in Peng (2011), Sandve et al. (2013), Stodden et al. (2014). We also established the tabular description of the model as proposed in Nordlie et al. (2009). We claim this revamped model now allows any researcher in computational neuroscience to run it and to obtain the exact same results as the ones described in the original article (see https://hal.inria.fr/hal-01109483 for complete description and https://github.com/rougier/Neurosciences/tree/master/basal-ganglia/guthrie-et-al-2013 for source code). Furthermore, the new description, as well as the new figures, allow anyone to rewrite the model using a different language, tools or software.

However, the whole process took us approximately 3 months. This is hardly acceptable for the reproduction of a computational model that should be straightforward.

Unfortunately, this is not an isolated case. If computer science offers a large set of tools for prototyping, writing, running, testing, validating, sharing, and reproducing results, computational neuroscience still lags behind. In the best case, authors may provide the sources of their model as a compressed archive and feel confident their model is reproducible. But this is not exactly true. Buckheit and Donoho (1995) explained almost 20 years ago that, “*an article about computational result is advertising, not scholarship. The actual scholarship is the full software environment, code and data that produced the result.”* The computational part in computational sciences implies the use of computers, operating systems, tools, frameworks, libraries, and data. This leads to such a large number of combinations (taking into account the version for each components) that the chances to have the exact same configuration as one of your colleague are nearly zero. This draws consequences in our respective computational approaches in order to make sure models and simulations can be actually and faithfully reproduced. We have to enforce the rules proposed in the literature and editors have to make sure this actually happens. From a broader perspective, this singular experience raises also some questions about the whole publication process. If articles remain the best media to publish a research and to introduce a model, why can't we have associated resources for the actual code just like we can have Supplementary Material as a separate document? For example, it is quite surprising that there is still no official code repository associated with journals. Even a dedicated public account on github (or any similar website) would really help on this matter.

But more importantly, given the quality of the new tools available today, it may be time to envisage new formats for communicating computational researches. For example, interactive documents could allow to replay a simulation and to modify parameters while reading the description of a model or a simulation. The IPython notebook is a serious candidate in that direction and could soon become a new way to exchange knowledge. It has been recently highlighted on Nature (Shen, 2014) and it is already widely used for teaching and writing books. Such new formats would definitely help authors, reviewers, readers and ultimately, Science.

## Conflict of interest statement

The authors declare that the research was conducted in the absence of any commercial or financial relationships that could be construed as a potential conflict of interest.
